# Genetic considerations in cerebral small vessel diseases

**DOI:** 10.3389/fneur.2023.1080168

**Published:** 2023-04-24

**Authors:** Riwaj Bhagat, Sandro Marini, José R. Romero

**Affiliations:** ^1^Department of Neurology, Boston Medical Center, Boston University School of Medicine, Boston, MA, United States; ^2^NHLBI’s Framingham Heart Study, Framingham, MA, United States

**Keywords:** cerebral small vessel diseases, stroke, lacune, white matter hyperintensities, microbleeds, perivascular spaces

## Abstract

Cerebral small vessel disease (CSVD) encompasses a broad clinical spectrum united by pathology of the small vessels of the brain. CSVD is commonly identified using brain magnetic resonance imaging with well characterized markers including covert infarcts, white matter hyperintensities, enlarged perivascular spaces, and cerebral microbleeds. The pathophysiology of CSVD is complex involving genetic determinants, environmental factors, and their interactions. While the role of vascular risk factors in CSVD is well known and its management is pivotal in mitigating the clinical effects, recent research has identified novel genetic factors involved in CSVD. Delineating genetic determinants can promote the understanding of the disease and suggest effective treatments and preventive measures of CSVD at the individual level. Here we review CSVD focusing on recent advances in the genetics of CSVD. The knowledge gained has advanced understanding of the pathophysiology of CSVD, offered promising early results that may improve subtype identification of small vessel strokes, has led to additional identification of mendelian forms of small vessel strokes, and is getting closer to influencing clinical care through pharmacogenetic studies.

## 1. Introduction

Cerebral small vessel disease (CSVD) has covered enormous interest as a major contributor to stroke and dementia, serving as potential target for risk stratification, prevention, and treatment. The term CSVD broadly sums up pathological processes affecting small vessels of the brain including small arteries, arterioles, capillaries, and small veins (1). Although the small vessels are difficult to image and investigate *in vivo*, technologic advances in neuroimaging have allowed qualitative and quantitative assessment of the effects caused by CSVD, which have been accepted as biomarkers of CSVD. The Standards for Reporting Vascular Changes on Neuroimaging (STRIVE) study defined magnetic resonance imaging (MRI) markers of CSVD as lesions of presumed vascular origin such as recent small subcortical infarct, lacune, white matter hyperintensities (WMHs), enlarged perivascular spaces (PVS), and cerebral microbleeds (CMBs). Recent subcortical infarcts, if symptomatic, are referred to as lacunar stroke, lacunar infarct, lacunar syndrome or small vessel stroke (SVS), while asymptomatic infarcts are labeled as “covert” or silent brain infarct (SBI) (2).

CVSD has been classified into various subtypes based on the pathology and the presumed underlying etiology, as summarized in Table 1. In the hypertension-related subtype, blood vessels undergo changes such as lipohyalinosis, wall thickening, lumen narrowing, microaneurysm and micro atheroma formation (1). In the cerebral amyloid angiopathy (CAA) subtype, amyloid fibrils deposit in the vessel wall and can be associated with fibrinoid necrosis, granulomatous inflammation, focal wall rupture and blood extravasation (3). These pathologies lead to broad clinical manifestations, including ischemic stroke, intracerebral hemorrhage (ICH), cognitive impairment, dementia, depression, and gait disturbance (4).

**Table 1:** Classification of cerebral small vessel diseases (Adapted from Pantoni et.al with permission).

Types	Description	Associated disease
Type 1	Arteriolosclerotic small vessel diseases	Hypertension, Diabetes mellitus
Type 2	Sporadic and hereditary cerebral amyloid angiopathy related small vessel diseases	
Type 3	Inherited or genetic small vessel diseases distinct from amyloid angiopathy	Cerebral Autosomal Dominant Arteriopathy with Sub-cortical Infarcts and Leukoencephalopathy (CADASIL) Cerebral autosomal recessive arteriopathy with subcortical infarcts and leukoencephalopathy (CARASIL) Mitochondrial encephalomyopathy, lactic acidosis, and stroke-like episodes (MELAS) Fabry’s disease Hereditary multi-infarct dementia of the Swedish type Hereditary cerebro-retinal vasculopathy Hereditary endotheliopathy with retinopathy Nephropathy and stroke *COL4A1* mutations associated small vessel diseases
Type 4	Inflammatory and immunologically mediated small vessel diseases	Wegner’s granulomatosis Churg-Strauss syndrome Microscopic polyangiitis Henoch-Schönlein purpura Cryoglobulinaemic vasculitis Cutaneous leukocytoclastic angiitis Primary angiitis of the central nervous system Sneddon’s syndrome Infectious nervous system vasculitis Connective tissue disorder associated nervous system vasculitis such as systemic lupus erythematosus, Sjögren’s syndrome, rheumatoid vasculitis, scleroderma, and dermatomyositis
Type 5	Venous collagenosis	
Type 6	Other small vessel diseases	Post-radiation angiopathy, Non-amyloid micro-vessel degeneration in Alzheimer’s disease

Several mechanisms have been implicated in these lesions, such as chronic hypoperfusion of the white matter secondary to luminal narrowing of the small blood vessels with disrupted cerebral autoregulation (5–7), blood–brain barrier dysfunction (8), PVS dysfunction (8), and focal subclinical inflammation (9). However, much remains to be elucidated in the pathophysiology of various subtypes of CSVD, which likely invokes the interplay of genetic and environmental factors. Thus, the study of genetic determinants of CSVD has covered major interest to help advance understanding of the role of genetic factors in relation to CSVD and related outcomes. As a complex group of disorders, most CSVD cases likely involve multiple genetic variants and gene–environment interactions, while a minority of cases are attributable to single gene mutations.

Epidemiological methods exploring genomics of complex diseases have advanced understanding of genetic contributions to CSVD. In this review we consider contributions from Genome-Wide Association Studies (GWAS), Multi-Trait Analysis of Genome-wide association summary statistics (MTAG), Transcriptome-Wide Association Studies (TWAS) and Mendelian Randomization (MR) analysis, aiming to provide the reader an updated review of genetic determinants of CSVD. While we recognize that the studies discussed are heterogeneous, represent various study designs, different samples and address different underlying disorders, we present an overview of current updates and discuss relevant clinical considerations where applicable. Our review aims at providing a multidisciplinary and comprehensive synthesis of all CSVD manifestations to give the reader an overview of the biological mechanisms underlying such a pleiotropic and complex disorder.

## 2. Epidemiology of different types of cerebral small vessel diseases

### 2.1. Prevalence and incidence

Epidemiological studies set the stage to understand the relevance of CSVD in population and hospital-based samples. Following current literature, we present data on CVSD by primarily identifying the single biomarkers of the disease and following definitions based on the STRIVE criteria (2).

#### 2.1.1. Community-based samples

##### 2.1.1.1. Silent (covert) brain infarcts

In populations of European ancestry, the overall prevalence of SBI has been reported to range between 11–28% (10, 11). In these studies the prevalence increased with advancing age, from 8% in individuals aged 60–64 years, to 35% among those aged 85–90 years. SBI was more frequent in women (23% versus 17% in men), and strongly related to hypertension, higher systolic and diastolic blood pressure, pulse pressure, cigarette smoking (12), and history of prior stroke.

In samples of Asian race and ethnicity, the overall prevalence of SBI was 10–16% (13). The prevalence also increased with age, from 8% in those aged 35–39 years to 45% in individuals aged 70–80 years. These studies suggest higher prevalence of SBI in older age among Asian groups compared with European groups. It is important to note, however, that these studies include individuals predominantly of older age among Asian groups compared with European groups.

##### 2.1.1.2. Lacunes

Lacunes have higher prevalence among Asian individuals (15–25%) (13–15) than European individuals (7%) (16). The prevalence of lacunes increased with age, from 5.3% in individuals aged 35–39 years to 41% in those aged 70–80 years. Lacunes were more frequent in men (22%) than women (10%), and were associated with hypertension, higher systolic and diastolic blood pressure, and diabetes.

##### 2.1.1.3. White matter hyperintensities

White matter hyperintensities had the highest prevalence among all CSVD markers, reaching 92% in European elderly samples (mean age ≥ 50 years), and higher in European women and individuals with advanced age (17). Confluent WMHs may show brain topography variations, reported more frequently noted in subcortical than periventricular regions. The frontal lobe was more affected than the temporal, parietal and occipital lobes in a prior study (17), but their distribution may depend on the underlying CSVD subtype. For instance, posterior distribution in parieto-occipital regions may be more frequent in CAA (18). Asian individuals had a prevalence of WMHs as high as 81%, which was noted in a specific high-altitude living Tibetan population (19). Across other Asian populations, there was a wide overall prevalence of WMHs ranging from 2.1 to 65% (14, 15). Prevalence also increased with age, from 32% in individuals aged 35–39 years to 90% among those aged 70–80 years, and was associated with hypertension and systolic blood pressure.

##### 2.1.1.4. Perivascular spaces

PVS visible on brain MRI are detected in most individuals, thus it is not their presence but the burden of PVS that seems to be clinically relevant. In European descendants the prevalence of moderate and severe PVS in the centrum semiovale was 15 and 3%, respectively, while in the basal ganglia was 9 and 1%, respectively (20). The overall prevalence of PVS in Asian populations seems higher, reported previously between 17–72% (13, 21). Risk factors including age, hypertension, higher systolic blood pressure and higher fasting glucose were associated with PVS (13). In both population-based and hospital-based samples, there was independent association of PVS (basal ganglia and centrum semiovale) with WMHs, advancing age, and history of SVS (22, 23).

##### 2.1.1.5. Cerebral microbleeds

Framingham Heart Study participants had 9% prevalence of CMBs, representing a general population in the United States (24). European population-based studies reported overall prevalence ranging from 6.4–15% (25, 26). Asian population-based studies reported a prevalence ranging from 3–37% (27, 28). Similar vascular risk factors like age, hypertension, higher systolic and diastolic blood pressure, and smoking were associated with the presence of CMBs. Additionally, male sex and lower low density lipoprotein cholesterol were associated with CMB presence (13, 24).

#### 2.1.2. Hospital-based samples

In contrast to community-based studies, patients attending a hospital or clinic, have a higher prevalence of CSVD markers. For instance, a study from Singapore compared memory clinic-based versus community-based population samples and found that the prevalence of lacunes was 28% vs. 18%, CMBs 42% vs. 36.5%, WMHs was 3.5% vs. 2%, and PVS was 17% vs. 7%, respectively (21). Similarly, higher prevalence of SVS (46%), CMBs (53%), and WMHs (93%) was observed in a hospital-based registry in Taiwan. Age and hypertension were among the major associated risk factors (29).

The presence of CSVD markers increases the risk of mortality by four-fold compared to healthy cohorts. Stroke, dementia, and ICH are all linked to CSVD, with reported prevalence in CSVD patients of 57%, 38–50%, and 1–44%, respectively (30, 31).

The above studies indicate that there is a spectrum of manifestations of CSVD with variations across ethnic and racial groups, higher prevalence in clinical samples than population-based samples, but strongly associated with age and hypertension regardless of the sample studied.

The observation of racial and ethnic variations is highly relevant as genetic factors may account for such differences. For instance, genetic studies have identified a non-synonymous single nucleotide polymorphism (rs2230500) in a member of the protein kinase C family (PRKCH), which showed a significant association with SBI and cerebral hemorrhage in Asians. In contrast, the minor allele frequency of this genetic variant is rarely found in European descendants (32, 33) Further studies are warranted to study the genetic variations among different ethnic groups and its risk for the CSVD.

## 3. Genetics of cerebral small vessel diseases

Although traditional vascular risk factors play a significant role in the onset and progression of CSVD, the precise etiology is still unknown because not all people who have those risk factors develop severe CSVD and the disease can develop in the absence of the vascular risk factors (34). It is likely that complex interactions between genetic and environmental factors are implicated in the development of clinical phenotypes in CSVD. Genetic contributions to CSVD were initially identified by studies showing high heritability of CSVD markers. The strongest heritability was demonstrated for WMHs in offspring and twin studies, ranging from 55 to 71% (35, 36), while for PVS and SVS were 59 and 48%, respectively (37). Clinical primary ICH (mostly caused by CSVD) also has high heritability, six-fold higher with a positive family history [odds ratio (OR) 6.3, 95% confidence interval (CI) 1.8–22] (38).

Many advances have taken place in genetic methods both at the individual and population levels. In what follows, we focused our discussion on the recent advancements in the genetics of both monogenic and sporadic forms of CSVD and discuss general aspects of specific genetic methods.

### 3.1. Monogenic forms of cerebral small vessel diseases

Monogenic (familial or Mendelian) forms of CSVD consist of single-gene disorders causing CSVD. Cerebral autosomal dominant arteriopathy with subcortical infarcts and leukoencephalopathy (CADASIL) is the most common and best characterized monogenic form of CVSD with a prevalence of 5 per 100,000 in Northern England (39). CADASIL is caused by mutations in the *NOTCH3* gene on chromosome 19q12 (40). The epidermal growth factor-like repeat group in these mutations is an important modifier of the age of onset for first stroke and CVSD imaging markers (41).

Other less common but well characterized diseases caused by monogenic mutations include *COL4A1, COL4A2, TREX1, HTRA1, ADA2, FOXC1, FOXF2,* and *GAL*. Additionally, *CTSA* has been recently discovered as a causal gene of a monogenic form of CSVD (42, 43).

Several clinical monogenic forms of ICH have also been described such as hereditary cerebral hemorrhage with amyloidosis syndrome associated with *APP* gene, whose clinical phenotype is characterized by development of lobar ICH and neuroimaging features such as WMHs and superficial siderosis (42). Genetic variants in the *MTHFR*, *IL6* and *TNF* genes have also been associated in both lobar and non–lobar ICH (44–46).

The pathophysiology and inheritance pattern of the monogenic disorders associated with these genes are summarized in Table 2.

**Table 2:** Pathophysiology of common monogenic forms of CSVD.

Disease	Genes	Chromosome Loci	Inheritance	Pathophysiology
CADASIL	*NOTCH3*	19q12	AD	Mutant *NOTCH3* transmembrane protein multimerizes to form the aggregates. Further deposits of matrix proteins such as tissue inhibitor of metalloproteinases-3, vitronectin, and latent Tumor Growth Factor (TGF)-beta binding protein-1 (LTBP-1) accompany NOTCH3 protein aggregates. These osmophilic granular deposits activates TGF-beta and contributes to vessel degeneration (47).
CARASIL	*HTRA1*	10q25	AR	Loss of protease *HTRA1* cleavage of LTBP-1 results in impaired signaling of TGF-beta with overall effects on pericyte proliferation and protein accumulation in vessel wall, metalloproteinase activity, blood–brain barrier permeability and dysfunctional cerebral blood flow (42).
COL4	*COL4A1/*2	13q34	AD	Mutation of Glycine residue of heterotrimers (alfa 1alpha1alpha2) of *COL4A1/*2 form mutant heterotrimer which impairs the structural components of basement membrane, cell-matrix, and cell–cell communication.
FOXC1/2	*FOXC1/2*	6p25		Mutation of *FOXC1/2* affects the blood–brain barrier, blood vessel stability and endothelial proliferation (48).
RCVL	*TREX1*	3p21	AD	Affects TREX1 3–5 DNA exonuclease involved in clearing cytosolic nucleic acids (49).
FD	*GLA*	Xq22	XL	Deficiency of alfa-galactosidase A leads to intracellular accumulation of neutral glycosphingolipids in vascular endothelium and neurons. It causes inflammation fibrosis, stenosis, dilatation and hypoperfusion (50).
Homocysteinemia	*MTHFR*	1p36	AR	Loss of function of cystathione synthetase activity leads to accumulation of homocysteine, homocysteine and its derivatives rendering toxic effect on arterial wall and atherosclerosis (51).
Sickle Cell disease	*HBB*	11p15	AR	Mutation in the beta-globin gene disrupts the architecture and flexibility of red blood cell in deoxygenated environment which causes sickling and vaso-occlusive hypoperfusion (52).

CADASIL, Cerebral autosomal dominant arteriopathy with subcortical infarcts and leukoencephalopathy; CARASIL, Cerebral autosomal recessive arteriopathy with subcortical infarcts and leukoencephalopathy; COL4A1/2, Collagen alpha-1/2; FOXC1/C2, Forkhead box protein C1/C2; RVCL, retinal vasculopathy with cerebral leukodystrophy; FD, Fabry disease; GLA, galactosidase Alpha; AD, autosomal dominant; AR, autosomal recessive; XL, X-linked.

The age of symptomatic individuals from these monogenic variants varies: 44–60 years (mean age) for *NOTCH3* mutation (40), median age 13 years for *ADA2* mutation, 60 years for *HTRA1* mutation, and under 18 years for *COL4A1/2* mutation (43).

The most common clinical features include ischemic stroke in *NOTCH3, COL4A1*, and *ADA2* mutations; cognitive disorder in *NOTCH3, HTRA1, COL4A2*, and *CTSA* mutations; developmental delay in *COL4A1/2* mutations; psychiatric features in *CTSA, HTRA1* and *TREX1* mutations; seizures in *COL4A1/2* mutations; vertigo in *CTSA* mutations; and headache in *TREX1* mutations (43). Individuals with *COL4A1/2* mutations have a wide spectrum of phenotypes including porencephaly, infantile hemiparesis, dementia, ICH, intracerebral aneurysms, ocular abnormalities (Axenfeld-Rieger anomaly), cardiac and renal abnormalities. Axenfeld-Rieger anomaly, characterized by anterior segment dysgenesis, craniofacial dysmorphism and systemic manifestations, is also observed with mutations in *FOXC1*. *α GLA* mutation manifests as ischemic stroke, childhood neuropathy, heart failure, gastrointestinal symptoms, corneal opacities, hearing loss and angiokeratoma (42).

As for CVSD neuroimaging markers, WMHs was the most common in all monogenic variants (100% in *CTSA*) but less frequent in *COL4A2* and *ADA2* mutation carriers. CMBs and SVS were common in *HTRA1* mutations, ICH in *COL4A2* mutations, enlar ged PVS and cortical atrophy in *CTSA* mutations. A unique arch-shaped hyperintense lesion ‘arc sign’ was observed in the pons to the middle cerebellar peduncles in *HTRA1* mutations (43). Of note variants in some of these genes have been found in sporadic forms of CSVD, such as *NOTCH3* (53), *COL4A1/A2,* (54) *TREX1* (55), and Fabry’s disease variants (56).

As noted, a large list of monogenic disorders has been added to current literature thus presenting a confusing picture to clinicians. While there is no strict rule to apply in all cases, patients with monogenic forms of CSVD tend to have frequent coexistence of MRI markers (SBI, WMHs, CMB), with more severe manifestations. Clinicians should include assessment of family history to clarify possible mendelian patterns of inheritance, and suspect monogenic disorders particularly in young individuals, when severe manifestations are noted on brain MRI, or systemic findings raise suspicion. A detailed clinical history is key to elicit possible psychiatric or more subtle neurological manifestations, and physical examination should include a detailed cardiovascular exam and skin inspection. Before proceeding with genetic testing for these disorders, genetic counseling should take place so that patients can make an informed decision considering the potential implications of genetic testing in clinical care.

### 3.2. Sporadic forms of cerebral small vessel diseases

Sporadic forms of CSVD are one of the most prevalent age-related neurological diseases contributing to significant morbidity and mortality. Environmental factors are associated with sporadic CSVD, the strongest of which is hypertension, but multiple genetic variants may play a significant role in their pathophysiology (57). We discuss genetic studies using various methodologies including candidate gene-based studies, GWAS and TWAS identifying the association of genes with sporadic forms of CSVD. The list of chromosome loci and genes associated with WMHs, SVS and ICH is increasing, at present with no clear direct clinical benefit but considered to help identify relevant aspects of the pathophysiology and diagnosis of CSVD, and potential for discovery of treatment targets; a summary is presented in Table 3. We simplified and categorized the pathophysiology of these genetic variants broadly into three groups affecting cellular mechanisms, blood vessel wall and blood brain barrier (Table 4).

**Table 3:** Chromosomal loci and genes associated with sporadic forms of cerebral small vessel diseases.

Loci	Genes	CSVD phenotype	Type of study
1q22	*PMF1-BGLAP (intron), SLC25A44-PMF1-BGLAP*	SVS, ICH	GWAS/TWAS (58–60)
1q41	*KCNK2*	WMHs	GWAS (61)
1p22	*PKN2*	WMHs	GWAS (61)
2p21	*HAAO*	WMHs	GWAS (60)
2p16	*EFEMP1 (intron)*	WMHs	GWAS (60, 62)
2q33	*NBEAL1, WDR12-ICA1L, CARF-FAM117B-ICA1L-NBEAL1, ICA1L-WDR12-CARF-NBEAL1*	WMHs, SVS, ICH	GWAS/TWAS (58, 62, 63)
2q32	*CALCRL*	WMHs	GWAS (61)
2p21	*HAAO*	WMHs	GWAS (61)
2p33	*CARF1*	WMHs	GWAS (61)
3q27	*KLKL24*	WMHs	GWAS (61)
3p22	*ULK4*	SVS	TWAS (59)
5q14	*VCAN*	WMHs	GWAS (61)
5q23	*LOC100505841, LOX-ZNF474-LOC100505841*	WMHs, SVS	GWAS (59, 61)
6q25	*PLEKHG1, FOXF2-FOXQ1*	WMHs, SVS	GWAS (59, 64)
6q24	*VTA1-GPR126*	WMHs, SVS	GWAS (59)
8p23	*SGK223, TNKS, XKR6*	WMHs	GWAS (61)
10q24	*SH3PXD2A (intron), SH3PXD2A (intron), AS1*	WMHs, SVS	GWAS (59–61)
10p14	*ECHDC3*	WMHs	GWAS (61)
10q26	*HTRA1-ARMS2*	SVS	GWAS (59)
11p11	*SPI1-SLC39A13-PASMC3-RAPSN*	SVS	GWAS (59)
13q34	*COL4A1-COL4A2*	WMHs, ICH	GWAS (58, 62)
14q32	*EVL, CCDC88C*	WMHs	GWAS (61, 62)
14p22	*NID2*	WMHs	GWAS (61)
14q32	*DEGS2*	WMHs	GWAS (61)
14q22-q23	*PRKCH*	SVS	Candidate gene study (33)
15q22	*RASL12*	WMHs	GWAS (61)
16q24	*C16orf95, ZCCHC14*	WMHs, SVS	GWAS (59, 61, 63)
16q12	*SALL1*	WMHs	GWAS (61)
17q23	*ACE*	WMHs, ICH	GWAS/Candidate gene study (65, 66)
17q25	*WBP2 -TRIM65-TRIM47- MRPL38-FBF1- ACOX1, NEURL (intron), PDCD11 (intron)*	WMHs	GWAS (59, 60, 67)
17q21	*C1QL1, NMT1*	WMHs	GWAS (61, 62, 68)
18p11	*ZBTB14-EPB41L3*	SVS	GWAS (59)
19q13	*APOE*	ICH	Candidate gene study (69)
22q12	*MN1*	WMHs	GWAS (61)
23p11	*TIMP1-TIMP2*	ICH	GWAS (70)

CVSD, cerebral small vessel diseases; SVS, small vessel stroke; WMHs, white matter hyperintensities; ICH, intracerebral hemorrhage; GWAS, Genome-Wide Association Studies; TWAS, Transcriptome-Wide Association Studies.

**Table 4:** Pathophysiology of genes in sporadic form of cerebral small vessel diseases.

Pathophysiological groups	Genes	Pathophysiology
Cellular mechanism	*TRIM65, TRIM47, FBF1*	Regulation of the apoptosis (67).
	*WBP2*	Regulates the conversion of DNA to RNA (transcription) (67).
	*ACOX1*	Affects the peroxisomal fatty oxidation pathway and involved in adrenoleukodystrophy (42).
	*NBEAL1*	Vesicle trafficking, membrane dynamics, receptor signaling, RNA processing, signal transduction and cytoskeleton assembly (71).
	*ZCCHC14*	Roles in nucleic acid binding and regulation of DNA transcription (72).
	*WDR12/ICA1L, MRPL38*	Encodes mitochondrial ribosomal protein essential for oxidative phosphorylation (73).
	*ECHDC3*	Involved in fatty acid biosynthesis (74).
	*KCNK2*	Encodes Twik-related K+ channel 1 (TREK1) of potassium channel expressed throughout the central nervous system (75).
	*SLC25A44*	Catabolism of branched chain amino acids in brown adipose tissue and mediates relationship between metabolic disease and SVS (76).
	*ULK4*	Encodes serine/threonine-protein kinase and involved in cell proliferation and differentiation, apoptosis, embryonic development, and myelination (59)
	*ZBTB14*	Encodes zinc finger transcription factor and promotes apoptosis.
	*EPB41L3*	Encodes membrane protein and promotes apoptosis (59).
	*PRKCH*	Encodes protein kinase C η (PKCη), a serine-threoine kinase, involved in progression of atherosclerosis (32).
Blood vessel wall	*EFEMP1*	Encodes for an extracellular matrix glycoprotein which affects the vessel development and matrix metalloproteinase activity (62).
	*EVL*	Regulates sprouting angiogenesis *via* vascular endothelial growth factor receptor-2 internalization and signaling (77).
	*COL4A2*	Encodes collagen of basement membrane.
	*PLEKHG1*	Role in vascular endothelium reorientation and responses to mechanical stress in endothelial cells (64).
	*VCAN, NID2, EFEMP1, COL4A2*	Encodes for the extracellular matrix proteoglycan (Matrisome protein) and affects the integrity of vessel wall (61).
	*LOX-ZNF474-LOC100505841*	Regulates vascular extracellular matrix (59).
	*SLC39A13*	Encodes transmembrane protein with roles in zinc transport found in connective tissue and blood vessels (78).
Blood brain barrier	*FOXF2-FOXQ1*	Regulation of pericyte differentiation and function of blood brain barrier (79).
	*GPR126*	Activated by collagen type IV and binds laminin-211 and affects integrity of blood brain barrier and myelination (80).

#### 3.2.1. Candidate gene-based study

A gene is referred to as a candidate gene if its protein product suggests the possibility of the disease (a biological candidate) or if it is in a region of the chromosome that has been associated with the disease (positional candidate). Candidate gene association studies examine the genetic variation within a small set of pre-specified genes that are associated with disease and help clarify whether the gene is involved in the disease and possible mechanisms involved in the association (81). Some insight has been gained through candidate gene studies addressing samples with clinical stroke attributed to CSVD. For instance, a case–control study in a Chinese population with a candidate gene association approach suggested that a polymorphism in loci 14q22-q23 (*PRKCH*, rs2230500) was associated with both ischemic stroke (OR1.31, 95% CI 1.08–1.60), specifically SVS (crude OR 1.40) and intracerebral hemorrhage (OR 1.94, 95% CI 1.21–3.10). The *PRKCH* gene polymorphism was found to be specific to Asian populations (33). A prior meta-analysis reported that a polymorphism in locus 17q23 (*ACE*, rs4646994) was associated with ischemic stroke (OR 1.37, 95% CI 1.22–1.53), intracerebral hemorrhage (OR 1.76, 95% CI 1.16–2.66) and WMHs in Asian populations (66, 82). Biffi and colleagues found that loci 19q13 (*APOE2*, *APOE4*) was associated with both hypertension and amyloid related ICH (69). The *APOE* gene has three allelic variants defined by two SNPs, rs429358 and rs7412, known as *APOE*-ε2, *APOE*-ε3, and *APOE*-ε4. *APOE*-ε4 was found to double the risk for lobar ICH in subjects of Caucasian ancestry (69). In an additional study of white individuals from the UK presence of any *APOE* ε2 alleles was associated with all ICH (lobar and non-lobar) and with lobar ICH but not with deep only ICH. The *APOE* ε4 allele was associated with lobar ICH and with finger-like projects on head CT, a marker considered to represent probable CAA (83). However, ethnic and racial differences have been suggested in the relation of *APOE* genotype and ICH. In a large meta-analysis including 13,124 participants *APOE* ε2 was associated with lobar ICH (OR 1.49; 95% CI 1.24–1.80) as was *APOE* ε4 (OR 1.51; 95% CI 1.23–1.85). In Hispanic and black participants, however, no associations were found. Analyses using propensity score matching accounting for hypertension burden showed a modest association of *APOE* ε4 with lobar ICH risk among Hispanics but not in black participants. In addition, neither *APOE* ε2 nor ε4 was associated with non-lobar ICH risk in any race/ethnicity (84). Further, a study of 907 cases of lobar ICH and 2,660 controls showed that both *APOE* ε2 and *APOE* ε4 were associated with lobar ICH among white participants (OR 1.5 95% CI 1.1–2.0, and OR 2.0 95% CI 1.5–2.6, respectively), but in black participants neither genotype was associated with lobar ICH. In contrast, hypertension was a significant risk factor for lobar ICH in all three racial and ethnic groups (85).

Additional candidate gene studies have also linked *COL4A1, COL4A2*, *TIMP-1* and *TIMP-2* genes with non-lobar ICH in specific populations (70). Similarly, variation in the *ACE* gene was found to increase risk for non-lobar ICH only in populations of Asian ancestry, possibly through a mechanism involving hypertension (65).

These studies suggest that the underlying etiology and genetic architecture of lobar ICH may vary according to racial and ethnic groups.

The complexity of the relations of *APOE* genotype with phenotypes considered to represent subtypes of ICH is further illustrated in its relation to cortical superficial siderosis (cSS). In a recent meta-analysis including large samples from memory clinic cohorts (*n* = 2,587), symptomatic CAA cohorts (*n* = 402), and a population-based study (*n* = 1,379), the presence of *APOE* ε4 alleles was associated with cSS but only among patients from memory clinics (OR 2.10 95% CI 1.11–3.99), while presence of *APOE* ε2 alleles showed an association in patients from memory clinics and those from symptomatic CAA cohorts (OR 2.42 95% CI 1.48–3.95), a relation that was stronger when cSS was disseminated. The specific genotypes showing consistent and strong associations were the *APOE* ε2/ε2 and *APOE* ε2/ε4 genotypes (86). These studies did not assess the role of racial or ethnic groups in these associations, but suggest that the clinical context where they are assessed is also relevant.

#### 3.2.2. Genome wide association studies

GWAS assess common genetic variations across the entire human genome to identify genetic associations with observable traits. GWAS can demonstrate gene–gene interactions and detect high-risk haplotypes or combinations of multiple single nucleotide polymorphisms (SNPs) within a single gene providing unbiased approaches to evaluate the polygenic model of disease (87). Over the past decade, multiple GWAS have identified multiple genes, chromosome loci and SNPs reaching genome-wide significance associated with CSVD phenotypes, including subclinical brain MRI manifestations and clinical stroke.

##### 3.2.2.1. GWAS of white matter hyperintensities

The Cohorts for Heart and Aging Research in Genomic Epidemiology (CHARGE) consortium GWAS identified six genes associated with WMHs on chromosome 17q25: *WBP2*, *TRIM65*, *TRIM47*, *MRPL38*, *FBF1*, and *ACOX1*. The study included primarily populations of European descent (67). Another GWAS, in addition to chromosome locus 17q25, identified genome wide-significant chromosome loci 10q24 and 2p21in European descent samples, and loci 1q22 and 2p16 in multiethnic samples to be associated with WMHs (60). The International Stroke Genetics (ISG) Consortium GWAS of predominantly European ancestry individuals studied shared genetic factors contributing to WMHs in community populations and stroke patients. The study found new independent loci associated with WMHs which include *EFEMP1*, *NABEAL1*, *EVL*, *C1QL1*, and COL4A2. *NABEAL1* (2q33), *EVL* (14q32), *C1QL1* (17q21), and *COL4A2* (13q34) were linked to WMHs in community populations and in stroke patients. Two of these associations influenced expression of nearby gene products *NBEAL1/ICA1L* and *EFTUD2* (62). An additional association with WMHs found by the ISG consortium was in the *PLEKHG1* gene (rs275350) located in chromosome 6 (64). Sargurupremraj et al. studied 50,970 predominantly European participants (*n* = 48,454) and an African American (*n* = 2,516) population from the CHARGE consortium and the UK Biobank. The study discovered an additional 23 independent loci associated with WMH volume at genome-wide significance (Table 3). This study found systolic, diastolic and pulse pressure were associated with WMHs even below the clinical threshold for hypertension and showed a strong association between genetically determined WMH burden and risk of ischemic stroke and ICH in the general population. Four loci, *NID2* (14q22), *VCAN* (5q14), *COL4A2* (13q34), and *EFEMP1* (2p16), were found to increase risk for WMHs independent of blood pressure (61). Additionally, Traylor et al. identified two new loci *FOXF2, FOXQ1* (6p25) and *VTA1-GPR126* (6p24) associated with WMHs. The risk loci associated with WMHs shared association with SVS, however, loci 17q25 was solely associated with WMHs. The risk loci explained 1.4% of overall heritability (59). Taken together these studies are slowly clarifying potential genes implicated in WMH, linking risk factors even at lower levels that considered clinically treatable, and revealing potential different mechanisms of disease underlying the complex trait represented by WMHs.

##### 3.2.2.2. GWAS of cerebral microbleeds

A recent genome-wide association study in a small sample (*N* = 454, 176 with CMB and 278 without CMB) found that 19 *APOE* polymorphisms at a suggestive significance level (*p* < 1 × 10–5) were associated with the presence of CMB and one with progression of CMB. The *APOE* ε4 genotype was independently associated with the presence and progression of cerebral microbleeds (OR 2.54, 95% CI 1.08–6.00 and OR 5.1, 95% CI 1.36–19.16), respectively (88).

An additional larger GWAS including 11 population-based cohort studies and 3 case–control or case-only stroke cohorts found one locus in the *APOE* region reaching genome-wide significance (rs769449; OR 1.33 95% CI 1.21–1.45) for any CMB, while *APOE* ε4 alleles were associated with strictly lobar CMB (OR 1.34, 95% CI 1.19–1.50) but not with mixed CMB. Variants that have been related to deep ICH, SVS and WMH were also associated with CMB (89). The authors highlight that genetic variants associated with CMBs may overlap with other CSVD markers and outcomes.

##### 3.2.2.3. GWAS of perivascular spaces

A recent large GWAS and whole-exome association studies including ~40,000 participants, from 21 population-based cohorts found 24 genome-wide significant risk loci. This study noted associations with centrum semiovale PVS at an early age suggesting that they could potentially play a role in early-life. Causal associations were found using MR studies, linking high blood pressure with basal ganglia PVS. This study identified novel loci that could be implicated in pathways involving processes such as extracellular matrix and membrane transport, which could be relevant as treatment targets (90).

##### 3.2.2.4. GWAS of small vessel Stroke

The MEGASTROKE consortium in a multi-ancestry GWAS identified two genes associated with SVS reaching genome-wide significance, *ZCCHC14* (16q24) and *WDR12/ICA1L* (2q33) (63). Likewise, the *PLEKHG1* gene located on chromosome 6 was associated with SVS (OR 1.09, 95% CI 1.00–1.19) (64). In a GWAS including acute stroke cases and controls of diverse ancestry Traylor et al., identified 3 novel loci associated with SVS including *ULK4* (3p22), *SPI1-SLC39A13-PASMC3-RAPSN* (11p11), and ZBTB*14-EPB41L3* (18p11). In a joint analysis considering WMHs, MTAG identified risk loci for SVS including loci *SLC25A44-PMF1-BGLAP* (1q22), *LOX-ZNF474-LOC100505841* (5q23), *FOXF2, FOXQ1* (6p25) and *VTA1-GPR126* (6p24). A mutation in the *SLC39A13* gene was associated with Ehlers-Danlos syndrome and vascular abnormalities (59).

##### 3.2.2.5. GWAS of intracerebral hemorrhage

In non-lobar ICH, GWAS studies identified variations in 1q22 (*SLC25A44, PMF1, BGLAP*) as a susceptibility locus for this subtype of ICH, typically attributed to hypertension related CSVD. The *APOE*-ε4 genotype may also be associated with ICH unrelated to CAA. Further, genetic variants in genes for *COL4A1*, *COL4A2, ICA1L, WDR12, CARF,* and *NBEAL1* have been involved in all manifestations of CSVD, including non-lobar ICH (54, 58). In lobar ICH, variants on the *CR1* gene were found to influence the severity of amyloid deposition and amyloid related ICH (91).

#### 3.2.3. Transcriptome-wide association study

Most GWAS discoveries are in non-coding regions of the human genome and have unknown functions. However, understanding their genomic regulatory roles in transcriptional activities provides relevant information about disease mechanisms and potential therapeutic implications. TWAS, which serves this purpose, is a gene-based association approach that investigates associations between genetically regulated gene expression and complex diseases or traits (92). Although there is paucity of TWAS studies in relation to CSVD, some have addressed clinical stroke with promising results in relation to stroke subtype identification, including those attributed to CSVD.

##### 3.2.3.1. TWAS in small vessel stroke

Although studies are in preliminary stages, some show promising results for identification of stroke subtype, which may help narrow the large proportion of strokes considered cryptogenic after thorough diagnostic testing, which in turn may have diagnostic, preventive and treatment implications. Jickling et al. conducted a study of ribonucleic acid expression profiles from blood to predict the etiology of cryptogenic stroke, leading to identification of 41 genes that distinguished small deep stroke as SVS and non-SVS (cardioembolic or artery-to-artery embolic stroke). The genes identified suggesting SVS with greater than 1.5-fold change included *HLA-DRB4, LAIR2, LGR6, LOC100132181, UTS2, PRSS23, RASEF, TGFBR3, CCDC114, FAM179A, OASL, CALM1, ALS2CR11, UBA7, PROCR, C18orf49, SCAND2, GBP4, TTC12, TSEN54, CCDC78, CCL2, CCL3/CCL3L1/CCL3L3, RUNX3* and *LAG3* (93, 94). These studies were based on diagnosis of SVS based on clinical and neuroimaging features but have important limitations to consider. Derivations of the gene predictors were based on large number of variables from a small sample, which is subject to false discoveries, and validation in independent cohorts is needed.

Traylor et al. performed TWAS in acute stroke cases and controls and identified genes for which genetically altered expression was associated with SVS. Genetically elevated expression of loci *SLC25A44* (1q22) *and CARF, FAM117B, ICA1L, NBEAL1* (2q33), and genetically decreased expression of locus *ULK4* (3p22) were associated with SVS. Importantly, some of these genes have potential implications in the relation of risk factors to stroke and clinical manifestations. Mutation in the *SLC25A44* gene was suggested to mediate the relation between metabolic disease and SVS. A mutation in the *ULK4* gene was further associated with acute aortic dissection and neuropsychiatric traits (59).

In addition to informing the pathophysiology of CSVD, TWAS may help identify protein biomarkers to guide treatment development in CSVD. Stamova et al. identified 79 new genes, in addition to 29 known genes, through a gene expression profile study from whole blood that predicted ischemic stroke with high sensitivity and specificity. This study suggested that genes for Factor V and thrombomodulin could be potential biomarkers for SVS (95). Another gene expression study identified an additional 40 gene profiles, which differentiated cardioembolic stroke from large vessel stroke (96).

Carmona-Mora et al., discovered 23, 188, and 43 differentially expressed genes in monocytes, neutrophils, and whole blood, respectively in SVS versus controls. Overall, gene expression of monocytes was down-regulated, and that of neutrophils was up-regulated. This study showed cell type-specific gene expression profiles in monocytes and neutrophils following ischemic stroke, affecting changes in downstream complex signaling pathways. Dysregulated blood cells in ischemic stroke may become biomarkers to further characterize SVS and serve as targets for treatment development in CSVD (97).

## 4. Epigenetic studies

Emerging data suggest the critical role of epigenetic risk factors in stroke causation. The understanding of epigenetic mechanisms like deoxyribonucleic acid methylation, histone post-translational modifications, and noncoding ribonucleic acids, which are essential for cell differentiation, growth and development, environmental adaptation, aging, and disease states, provides insight about stroke pathogenesis. For example, deoxyribonucleic acids methylation can be affected by folate levels which are influenced by dietary factors. Healthy balanced diet induces methylation in Long Interspersed Element (LINE) stabilizing genome in the peripheral blood cells (98), and hypomethylation of LINE is associated with risk of cardiovascular diseases. Likewise, diet modulated micro ribonucleic acid target site and hypermethylated *APOE* genes are associated with risk of stroke (99). Traylor et al. reported that 11 of 12 lead SNPs associated with SVS influenced deoxyribonucleic acid methylation (59). Nevertheless, while epigenetic drug targets may lead to interventions to mitigate the burden of stroke, further epigenetic study on CSVD is warranted.

## 5. Mendelian randomization

Epidemiological studies of cardiovascular risk factors and CSVD can be biased by reverse causation and confounding factors. MR helps to eliminate these biases aiming at identifying the causal association of single nucleotide polymorphisms and the trait or disease of interest. In European ancestry, MR analysis of MRI markers of CSVD identified positive associations of diastolic, systolic, and pulse pressure, type 2 diabetes, and ever smoking with SVS (59). There was casual association of diastolic more than systolic blood pressure, pulse pressure, body mass index, ever smoking, and lipid traits with WMHs (61, 100). MR analysis has highlighted that higher levels of blood pressure even below the definition of hypertension are associated with larger WMHs volume (61). This finding has supported new possible strategies of intensive blood pressure lowering in high-risk individuals even without hypertension (61).

## 6. Limitations of genetic studies

The heterogeneity of CVSD manifestations presents a challenge to determine its underlying genetic determinants. WMHs have significant phenocopies like demyelinating disease, remote injuries, migraine headache and leukodystrophies, which affect the interpretation of genetic associations with specific etiologies and heritability estimates. Thus, refined definition of CSVD phenotypes in MRI and beyond MRI markers might be necessary for accurate genetic study.

Many genetic studies have not adjusted for age and sex, and the possibility of bias exists with substantial differences between case and control populations (59). Genetic studies have predominant representation of European ancestry thus limiting the ability to extrapolate the conclusions to other ancestry groups. The pleiotropy of genes variants makes identifying a single pathological pathway challenging (61).

In addition, specific genetic studies have limitations inherent to the methodology. For instance, candidate gene studies frequently produce results that are not supported by later research because they tend to restrict candidate selection to pathways that are previously thought to be significant for the phenotype. One downside of GWAS is the rigorous and specified genome-wide significance thresholds which can lead to false-negative results (101). Another drawback is that disease susceptibility-related functional variants are rarely detected, necessitating additional work to confirm the significance with precise mapping and functional testing. For example, in the MEGASTROKE consortium GWAS, additional gene-based testing, VEGAS2, was used to confirm the association of the neighboring genes *ICA1L* and *WDR12* with SVS (63). MTAG analysis is based upon assumption that associated variants act on both traits of interest, which might not be the case for WMHs and SVS, as they reflect downstream effects of a shared common ancestor. Independent replication of the involvement of the gene of interest in the disease is therefore the gold standard to establish causation (59).

## 7. Future directions

The complex role of gene–environment interactions in the pathogenesis of CSVD should be emphasized. Gene variants, rather than causing the disease, might predispose to disease through its interaction with the environment. While it is challenging to study the context in which genetic variants lead to disease, identification of molecular or biochemical phenotypes of such variants might offer additional revealing insight. Further, well-powered studies with large samples are needed to unveil the small effects from gene–environment interactions. Additional methods show promise to advance the understanding of genetic determinants of CSVD. Examples include studies of genetic risk scores and genetic risk load studies (101, 102). Cell programming studies in cell cultures, where cells are directed into disease-specific cell types, might help identify functional effects of genetic variation in the development of CSVD (103). For instance, introducing specific genetic variants with great accuracy in cell cultures through clustered regularly interspaced short palindromic repeats (CRISPR)/Cas9 system (104). These studies add to our understanding of the pathophysiology underlying CSVD and stroke and provide the basis to prioritize the most likely biological-candidate genes for integration of functional and biological information, and potential identification of drug target genes and existent drug repurposing (63). Stroke genetic risk scores may be predictive of SVS and have potential for use in the management of patients (105). Recent studies have offered genetic proof for potential drug actions, identifying *F11, KLKB1, PROC, GP1BA, LAMC2,* and *VCAM1* as potential target genes. Some drugs are already being studied for their potential in treating stroke targeting *F11* and *PROC* variants (105).

The field of pharmacogenetics is advancing at a fast pace and likely to influence clinical care in the short term, including use of the medications most commonly used for prevention of CSVD related clinical events. The recent CHANCE-2 clinical trial found that genotype-guided treatment (e.g., identification of CYP2C19 loss of function allele) with ticagrelor and aspirin for minor small vessel strokes or high-risk transient ischemic attack resulted in a higher relative risk reduction of stroke re-occurrence at 90 days compared to using short term clopidogrel and aspirin (106). The effects were attributed to lack of metabolization of clopidogrel into its active ingredient among participants with CYP2C19 loss of function alleles (106).

A recent open-label, multicenter, controlled, cluster-randomized, crossover implementation study assessed a 12-gene pharmacogenetic panel (68). The study evaluated actionable drug–gene interaction test results for which a change to standard-of-care drug treatment would be considered. The study was not only feasible, but also found that use of the pharmacogenetic panel significantly reduced the incidence of clinically relevant adverse drug reactions. The most common index drugs were atorvastatin and clopidogrel, which are among the main treatments used in patients with CSVD related stroke.

In addition to help identifying treatment targets, genetic studies are needed to understand if specific genetic factors determine specific clinical manifestations of CSVD, for instance whether specific genetic variants determine that patients with CSVD are at higher risk to develop depression, gait disorder or cognitive impairment, or other specific symptoms.

Finally, it should be highlighted that studies need to be inclusive of multi-ethnic and under-represented populations. Studying genetic differences in these groups might provide more insights into disease mechanisms and are needed to increase generalizability of findings.

## 8. Conclusion

CVSD represent a group of disorders highly prevalent around the world and contributing to significant morbidity and mortality. Genetic studies have identified several risk genes associated with CSVD, helped to better understand its underlying pathogenesis, and are advancing the field to identify potential treatment targets. Further studies to address gene–environment interactions in the pathophysiology of CVSD, categorize high-risk individuals and evaluate genetic targeted therapies are promising directions toward effective management and prevention of CSVD. The knowledge about CSVD gained from the genetic studies has been remarkable and is likely to enter clinical practice over the ensuing years.

## Author contributions

RB, SM, and JR conceptualized, designed, and collected data. RB and SM wrote the manuscript. SM and JR supervised the research and manuscript writing. All authors have read and agreed to the published version of the manuscript.

## Funding

JR received funding from National Institute on Aging grants (R01 AG059725; AG008122; AG054076; K23AG038444; R03 AG048180-01A1); and the National Institute of Neurological Disorders and Stroke (R01-NS017950-37).

## Conflict of interest

The authors declare that the research was conducted in the absence of any commercial or financial relationships that could be construed as a potential conflict of interest.

## Publisher’s note

All claims expressed in this article are solely those of the authors and do not necessarily represent those of their affiliated organizations, or those of the publisher, the editors and the reviewers. Any product that may be evaluated in this article, or claim that may be made by its manufacturer, is not guaranteed or endorsed by the publisher.
